# insilicoSV: a flexible grammar-based framework for structural variant simulation and placement

**DOI:** 10.1093/bioinformatics/btaf594

**Published:** 2025-10-31

**Authors:** Enzo Battistella, Nick Jiang, Chris Rohlicek, Ilya Shlyakhter, Victoria Popic

**Affiliations:** Broad Clinical Labs, Cambridge, MA 02142, United States; Broad Institute of MIT and Harvard, Cambridge, MA 02142, United States; Broad Institute of MIT and Harvard, Cambridge, MA 02142, United States; University of California, Berkeley, Berkeley, CA 94720, United States; Broad Institute of MIT and Harvard, Cambridge, MA 02142, United States; Broad Institute of MIT and Harvard, Cambridge, MA 02142, United States; Broad Clinical Labs, Cambridge, MA 02142, United States; Broad Institute of MIT and Harvard, Cambridge, MA 02142, United States

## Abstract

**Summary:**

Structural variants (SVs) are key drivers of genetic variation and disease in the genome. Their discovery remains challenging, however, in large part due to the scarcity of validated SV callsets and comprehensive benchmarks, which are essential for method development and evaluation. The growing number of data-driven learning-based approaches for SV discovery, in particular, requires large, diverse, and well-balanced training datasets to achieve reliable performance. To address this need, SV simulation has served as a key tool for assessing method performance and training SV models. However, existing SV simulators only support a fixed and limited set of SV classes and do not provide fine-grained control over the placement of SVs within specific contexts of the genome. Here we present insilicoSV, a versatile framework for SV simulation, which models SVs using a simple and flexible grammar, allowing users to easily define standard and custom arbitrary genome rearrangements, as well as encode genome placement constraints. This design allows insilicoSV to naturally support new and bespoke SV types, such as the complex rearrangements of cancer genomes. In addition to grammar-based modeling, insilicoSV provides built-in support for 26 predefined SV types, placement of user-provided SVs, small variant simulation, streamlined workflows for the simulation of genome evolution and genome mixtures, read simulation, alignment, and visualization. These features enable the creation of comprehensive genomic datasets for a variety of downstream applications, such as in-depth benchmarking of alignment and variant calling methods, as well as training of data-driven learning-based approaches for SV detection.

**Availability and implementation:**

insilicoSV is available under the MIT license at https://github.com/PopicLab/insilicoSV and https://doi.org/10.5281/zenodo.17402009.

## 1 Introduction

Structural variants (SVs) make up the broad class of large-scale genome rearrangements, which can be categorized as simple (e.g. deletions, inversions, tandem duplications, insertions) or complex multi-breakpoint mutational events (e.g. inverted duplications and deletion-flanked inversions) (Collins *et al.* 2020). SVs are the greatest source of genetic diversity in the human genome (Beyter *et al.* 2021) and drive numerous medically-relevant phenotypes (Weischenfeldt *et al.* 2013; Merker *et al.* 2018; Mantere *et al.* 2019; Li *et al.* 2020). While many methods for SV calling and genotyping have been implemented to date (Cameron *et al.* 2019; Ahsan *et al.* 2023), accurate SV discovery remains an open problem and an active area of research.

Patterns (or signatures) of discordance derived from read alignments to a reference genome are typically used to localize and classify SVs. These patterns are a function of multiple domain variables, such as the SV type, size, zygosity, genome context, genome coverage, alignment methodology, and read sequencing technology (e.g. read length and error profiles). As such, the development and comprehensive characterization of SV methods requires the availability of SV benchmark datasets, which reflect the diversity of the underlying SV signatures found in the genome. Such datasets can allow us to identify and address failure regimes and biases in existing and novel methods. Balanced and diverse SV datasets are particularly crucial for training the emerging class of deep learning methods for SV discovery, such as Cue (Popic *et al.* 2023) and SVision (Lin *et al.* 2022). Beyond SV discovery, SV truthsets can be used to study the impact of SVs on the accuracy of read aligners and downstream small variant callers, which are known to suffer from systematic errors near SV breakpoints.

However, detecting and validating SVs in real samples is very challenging. As a result, high-confidence SV truthsets are scarce. The Genome in a Bottle (GAIB) HG002 sample (Zook *et al.* 2020), which includes SV annotations validated using multiple sequencing technologies, is one of the few high-confidence SV truthsets currently available for benchmarking. As a result, genome simulation has been an essential tool for method evaluation. To generate a simulated whole-genome sequencing (WGS) dataset with a known SV truthset: (i) a synthetic genome is first created based on a reference genome and a set of simulated target SVs and (ii) this genome is then used as a template for read simulation (for a desired coverage and sequencing technology). Although limited in scope (as it only captures modeled SVs rather than the true full complexity of variation in a real genome), this approach allows us to both fully control the composition of the dataset and generate large amounts of annotated data with diverse labels.

Although numerous methods have been proposed to date for SV simulation (Jeffares *et al.* 2017; Bolognini *et al.* 2020; Mu *et al.* 2015; Bartenhagen and Dugas, 2013; Xia *et al.* 2018; Dierckxsens *et al.* 2021), existing tools do not comprehensively support all known SV classes and provide only limited control over key SV features (e.g. placement in the genome and size). For example, SURVIVOR (Jeffares *et al.* 2017) supports a limited number of complex SV types and provides no control over the placement of SVs in the genome. RSVSim (Bartenhagen and Dugas, 2013), SVEngine (Xia *et al.* 2018), and VISOR (Bolognini *et al.* 2020) can confine variants to specific regions (e.g. by excluding regions from placement); however, they similarly lack support for many key SV classes and fine-grained placement control. No existing simulator can support all the complex SV classes described in a recent gnomAD release (Collins *et al.* 2020). Importantly, no existing method supports the simulation of custom rearrangements.

insilicoSV overcomes these limitations by combining, in a unified and versatile framework, support for a wide range of variants and fine-grained control over their key characteristics. By introducing a simple grammar to describe structural rearrangements, insilicoSV enables users to define and simulate any custom SV type. This extensibility is particularly important given our incomplete knowledge of the full spectrum of possible complex SVs in the genome. Importantly, it provides users with a mechanism to test hypotheses about an observed complex rearrangement pattern, which could be a novel SV class. Finally, similar to several other tools (e.g. Xia *et al.* 2018; Bolognini *et al.* 2020), insilicoSV implements pipelines to simplify downstream analysis, including the simulation of genome evolution and genome mixtures, read simulation, mapping, and visualization of SVs for short and long-read platforms.

The key features uniquely implemented in insilicoSV include:

- Largest catalog of known complex SV classes, including out-of-the-box support for all the SV types reported in gnomAD.
- Grammatical SV notation: simple syntax to describe rearrangement operations (e.g. inversions, translocations, duplications), which enables the simulation of custom SVs.
- Fine-grained genome placement control: SVs can be constrained within specific regions of interest (ROIs), such that either the entire SV or specific SV breakpoints overlap the desired region type; supported placement constraints include: full containment within the ROI, full containment of the ROI, exact breakpoint overlap with ROI boundaries, partial overlap, and blacklist regions.
- Fine-grained size simulation control: inter-breakpoint distances can be configured independently for multi-breakpoint complex SVs; lengths can be drawn from independent distributions or be derived based on the distance between specific SV breakpoints (e.g. a delINV can be configured such that the deletion length is a function of the inversion length).
- Modular SV definitions: users can define and configure any number of SV categories within the same genome by combining different attributes (e.g. SV type, SV size, and placement constraints).

Taken together, the unique capabilities of insilicoSV enable the design and creation of highly diverse and complex synthetic genomes, which are instrumental for advancing SV discovery methods and addressing a wide array of other downstream applications.

## 2 Features of insilicoSV

### 2.1 Framework overview

**Figure 1.**
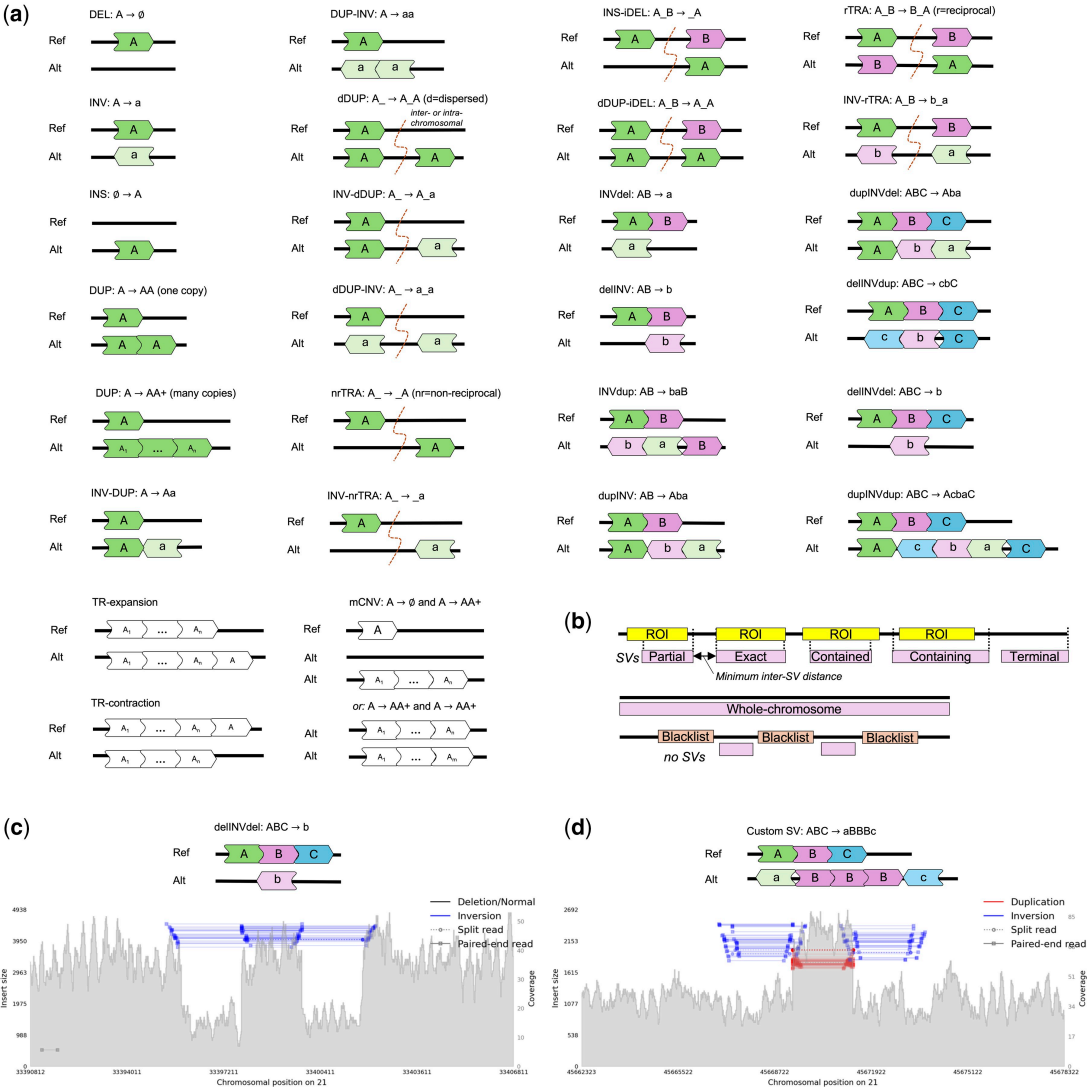
insilicoSV simulation features and output examples. (a) Diagrams of SV classes predefined in insilicoSV. These classes can be simulated directly by name or using the grammatical representation displayed for each class. Note: several symmetrical configurations have been omitted for conciseness (e.g. the INV-DUP A→aA structure). (b) Illustration of supported SV placement constraints. Given a list of ROIs, SV placement can be configured such that (i) one breakend is inside the ROI (partial), (ii) both breakends coincide with the ROI coordinates (exact), (iii) both breakends are contained within the ROI (contained), (iv) the ROI coordinates are contained within the SV breakends, (v) one breakend aligns with the end of the chromosome (terminal), (vi) the SV spans the entire chromosome (whole-chromosome), and (vii) no SV breakends occur in the ROI (blacklist). (c) Samplot (Belyeu *et al.* 2021) illustration of short-read alignments (showing the resulting coverage, pair orientations, and split reads) at the site of a delINVdel simulated with insilicoSV. (d) Samplot illustration of short-read alignments at the site of a custom SV simulated with insilicoSV using the provided SV grammar.

Given a reference genome and a set of user-provided configuration parameters, insilicoSV first generates a set of SVs that satisfy the constraints defined in the configuration and then transforms the reference genome into a new synthetic diploid genome with these SVs. The configuration parameters are specified as a YAML file and include the definition of one or more SV categories to simulate. The definition of each SV category includes the 4-tuple (*t*, *D*, *C*, *n*), where *t* represents the SV type (specified by name, e.g. DEL or invDUP; or as a rearrangement expression, e.g. ABC→AC; see Fig. 1a), *D* is a list of breakend distance ranges (specifying the minimum and maximum distance allowed between the SV breakends), *C* is a set of placement constraints (optional; see Fig. 1b), and *n* is the number of such SVs to simulate. The YAML file can also include global parameters (which apply to all SV categories), such as: a list of ROI files (in BED file format) to be used for SV placement and the minimum distance required between simulated SVs. SV categories can also be configured using a VCF file with existing SVs, which will be directly incorporated into the output genome using the exact type and breakpoint positions provided in the file. This mode allows users to create genomes based on SVs previously discovered in real data or to test a hypothetical transformation at a specific location.

To generate the SVs defined by each category, insilicoSV works in three stages. First, it instantiates the *n* SVs of type *t* by (i) sampling the distances between breakends from the corresponding entries of *D* uniformly at random (or deriving these distances based on the length of other intervals) and (ii) randomly assigning the SVs to either one or both haplotypes (for heterozygous and homozygous events, respectively). Next, it assigns the reference position of each SV breakend, randomly selecting from all the positions that satisfy the placement constraints *C* and ensuring that SVs do not overlap on the same haplotype (overlap across haplotypes can be enabled using a global placement parameter). Finally, it constructs the two synthetic output haplotypes, by editing the reference according to the SVs assigned to each haplotype. These two haplotypes can be coupled with read simulators to generate WGS datasets for downstream analysis. Figure 1c shows the short-read signature of the complex delINVdel event simulated with insilicoSV (using the provided short-read workflow).

### 2.2 Built-in SV library

insilicoSV provides built-in support for a library of 26 predefined SV types (allowing the user to simulate these SVs directly by name). This library covers a broad set of SVs, including simple SVs, dispersion-based SVs (e.g, dispersed duplications), flanked inversions, and tandem repeat expansions and contractions. For instance, all the SV types identified in gnomAD (Collins *et al.* 2020) are supported out-of-the-box. In addition to SVs, insilicoSV also supports small variants (e.g. SNPs), allowing for a comprehensive simulation of the full spectrum of variation in the genome. Schematics of all SVs included in the insilicoSV library are shown in Fig. 1a.

### 2.3 SV grammar

insilicoSV uniquely supports the simulation of SVs from structural rearrangement expressions that map reference alleles to alternative SV allele structures. This feature allows insilicoSV to support arbitrary custom rearrangements of the genome (which may not have an associated class name) and seamlessly extend to new SV types without requiring a change in the simulation software (e.g. Fig. 1d shows the short-read signature of a custom complex rearrangement). To that end, insilicoSV provides a simple grammar to define a set of operations on reference intervals, where reference intervals are represented as capital letters (e.g. A, B, or C) and the operations induced by the SV are represented as modifications of these letters or the order in which they appear. In particular: (i) an inversion of a reference interval is represented using the lowercase (e.g. the grammar rule A→a denotes a simple inversion of the reference allele A); (ii) a duplication of an interval is represented by multiple copies of the same letter (e.g. ABC→AABCC denotes a tandem duplication of A and C); a variable number of copies can be indicated using the ‘+’ symbol (with the number of copies drawn at random from a user-defined range); (iii) a translocation can be indicated by rearranging the order of the reference intervals (e.g. ABC→ACB denotes a translocation of B); (iv) a dispersion interval can be represented using the ‘_’ symbol, which allows other SVs to be placed in this interval (e.g. A_→A_A denotes a dispersed duplication of A); note: dispersions can be configured to be intra- or inter-chromosomal (e.g. A_B→B_A and the interchromosomal flag set to True will produce an inter-chromosomal reciprocal translocation); and (v) letters appearing only on the left or only on the right side of a grammar rule represent, respectively, deletions and novel insertions (e.g. A→AB represents an insertion of B, while ABC→AC represents a deletion of B); note: novel insertion sequences can be generated fully at random given a size range or sampled from a user-provided file containing custom sequences. The grammatical representation of an SV type provides a short string identifier unambiguously describing the rearrangement structure, which is particularly useful for the annotation of complex SV types with no standardized names. It also provides a simple way to refer to specific SV intervals, enabling a clear syntax for configuring their size distributions and placement constraints. In particular, placement constraints can be indicated grammatically using the ‘()’ anchor notation, which can be wrapped around a single or multiple consecutive reference alleles or left empty to constrain a single breakend (e.g. A(B)C→b allows the user to assign the inverted interval B to a specific ROI (e.g. a repeat type) flanked by t he deletions of A and C).

### 2.4 Context-aware SV placement

insilicoSV can be configured to embed SVs within specific types of regions of the genome, such as different genes or types of genomic repeat elements that highly correlate with and can mediate SVs (Mills *et al.* 2007; Niu *et al.* 2022; Sanchis-Juan *et al.* 2018; Gilbert *et al.* 2002; Gu *et al.* 2015). Since the genome context can significantly affect read alignments at SV-impacted loci, accounting for these associations in simulation and designing benchmark genomes that include SVs in different genome contexts is instrumental to fully characterizing the performance of downstream SV calling methods. For instance, repetitive genome regions will yield alignments with a reduced mapping quality and can otherwise distort the signals used to identify SVs. GIAB recently released stratifications for several reference genome versions (Dwarshuis *et al.* 2024), which bin genome regions based on mappability, complexity, GC content, ancestry patterns, repeat types, technical sequencing difficulty, and other key context features. The GIAB reference stratifications, RepeatMasker (Nishimura, 2000) tracks, or other genomic annotations can be provided directly to insilicoSV (in BED format) to enable context-aware placement. insilicoSV provides several ways to control the placement of SVs in relation to ROIs. For each variant category, the user can configure the types and sizes of ROIs onto which simulated variants can be placed, the SV breakends constrained to overlap the ROI, and the mode of overlap. The mode defines whether the SV breakends exactly coincide with the ROI, partially overlap it, fully contain it, or are fully contained within it. The mode can also be used to place SVs at the terminal regions of a chromosome (with one breakend corresponding to the chromosome start or end) or to simulate chromosome-scale events (entire chromosome gains or losses). For example, one can simulate deletions which exactly coincide with LINE-1 elements, dispersed duplications where either the origin or the target lies within one of several repeat types, or a deletion-flanked inversion where the deletion partially overlaps a specific type of repeat. An additional way to control SV placement is also to specify a set of blacklist regions with which no SV breakpoint may overlap and to specify a minimum distance between different SVs.

In order to place SVs under the specified placement constraints, insilicoSV follows a greedy heuristic, placing SV categories with the most restrictive constraints first. To that end, it places SVs in the following order: (i) SVs with fixed positions imported from a VCF file, (ii) SVs with breakpoints that must exactly match ROI boundaries, (iii) SVs with partial ROI overlap (i.e. when a single breakpoint must be in the ROI), (iv) SVs that must fully contain an ROI, (v) SVs that must be contained in an ROI, and (vi) all other unconstrained SVs.

### 2.5 Simulation output

The primary outputs of insilicoSV include a pair of FASTA files containing the two synthetic genome haplotypes, and a VCF file describing the simulated variants. Multi-breakpoint complex SVs are represented by multiple VCF records that can be linked using a unique identifier assigned to each SV. Additionally, each VCF record is annotated with the grammatical representation of the SV, as well as the specific symbol and operation it represents (required for interpreting complex events). insilicoSV also outputs a pair of PAF files, representing the correspondence between regions of synthetic haplotypes and of the reference. For each region comprising a synthetic haplotype, the PAF file provides the provenance of that region in the reference genome, the orientation relative to the reference, and the identifier of the relevant SV (if any). The PAF file can be used for lifting over coordinates between the synthetic genome and the reference—for example, to evaluate the accuracy of mapping reads simulated from the synthetic genome to the reference—and for visualizing the simulated SV transformations using tools such as SVbyEye (Porubsky *et al.* 2025). A statistics file is also produced, summarizing the counts and sizes of the simulated variants and the total length difference of each chromosome.

### 2.6 Workflows

The insilicoSV package includes customizable WDL pipelines that support: (i) the simulation of a single synthetic genome using insilicoSV, (ii) the simulation of linear genome evolution across multiple time points (with genomes produced for each intermediate time point), (iii) the simulation of a mixture of related genomes resulting from a branched evolutionary lineage tree (e.g. cancer subclones), (iv) read simulation from the generated synthetic genome(s), (v) read alignment, and (vi) SV visualization. The pipelines can be configured to simulate reads from multiple sequencing platforms, including Illumina, PacBio and Oxford Nanopore Technologies. Short-read simulation is implemented using DWGSIM (Homer, 2010), while long-read simulation uses PBSIM3 (Ono *et al.* 2022). Read alignment is performed using minimap2 (Li, 2018) with platform-specific presets. SV visualization is implemented using IGV-reports (Thorvaldsdóttir *et al.* 2013) (in WDL) and Samplot (Belyeu *et al.* 2021) (in the accompanying demo Jupyter notebook).

### 2.7 Case study

To illustrate a key use case of insilicoSV, we used its context-aware SV placement feature to evaluate SV calling across different genome contexts. To that end, we used insilicoSV to generate three synthetic genomes, each containing 500 deletions of size 1–10 kbp (with at least 1kbp distance between each SV), placed in (i) unique/non-repetitive regions of GRCh38 chr1—we used the “blacklist” placement feature of insilicoSV and RepeatMasker tracks to guarantee that both SV breakpoints do not fall within any RepeatMasker intervals, (ii) LINE-1/L1HS repeat regions of GRCh38—we used the ”exact” overlap mode to place the deletion breakpoints precisely at the LINE-1/L1HS interval boundaries, and (iii) ALR/Alpha satellite regions of GRCh38 chr1—we used the ”containing” overlap mode to place both breakpoints within this repeat type. For each genome, we generated Illumina short reads and PacBio HiFi long reads at 30x coverage. Supplementary Fig.1a shows the difference in short-read and long-read alignments at homozygous deletion sites within each context. As expected, given the length of each repeat type and the length of the reads, we observe that (i) some fraction of short reads (originating from other copies of the deleted sequence) map within the deleted L1HS interval and (ii) both short and long reads (originating from other copies of the deleted sequence) map within the deleted interval in the much longer ALR/Alpha repeat (with low mapping quality split-read alignments/discordant read pairs spanning the deletion; as well as spurious split-read alignments reported within the deletion locus in long reads). Supplementary Fig.1b shows the precision and recall (computed using Truvari (English *et al.* 2022)) of four popular SV callers in each genome context: Manta (Chen *et al.* 2016) and DELLY (Rausch *et al.* 2012) using short reads and Sniffles2 (Smolka *et al.* 2024) and SVIM (Heller and Vingron, 2019) using long reads (all run with default parameters). Notably, while short-read and long-read methods perform well in unique regions, the recall of short-read callers drops in the L1HS genome by more than 15%, and the performance of all callers is low in the ALR/Alpha repeat (both long-read methods achieve a precision below 25% and a recall below 50%).

## Supplementary Material

btaf594_Supplementary_Data

## Data Availability

Source code, test data and documentation are freely available for download at https://github.com/PopicLab/insilicoSV and https://doi.org/10.5281/zenodo.17402009.
