# Supplementary material for: insilicoSV: a flexible grammar-based framework for structural variant simulation and placement
Source: Bioinformatics. 2025 Oct 31;41(11):btaf594. doi: 10.1093/bioinformatics/btaf594 (PMC12603351; doi:10.1093/bioinformatics/btaf594)
Supplement: btaf594_Supplementary_Data [file btaf594_supplementary_data.pdf]

Supplementary Data

Supplementary Figures

a

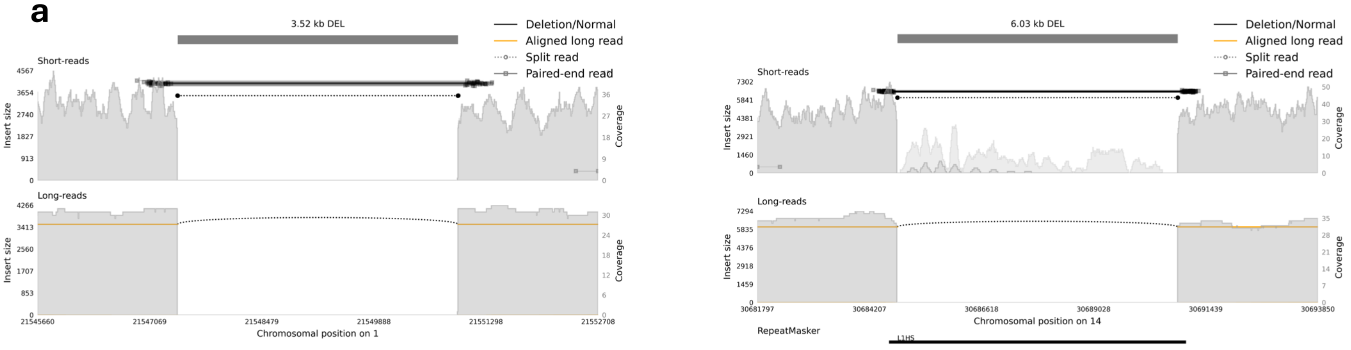

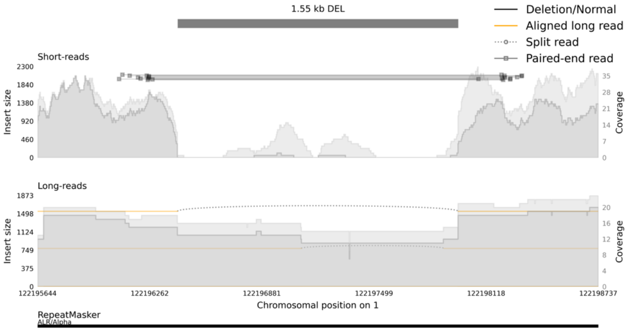

b

	Unique (blacklist)		L1HS (exact)		ALR/ Alpha (contained)	
	Prec	Rec	Prec	Rec	Prec	Rec
Manta	100	93.2	100	70.4	100	3.8
DELLY	99.8	94.8	98.5	77.6	77.6	10.4
Sniffles2	99.0	99.6	100	100	24.4	36.0
SVIM	99.0	99.6	100	100	24.9	36.8

Supplementary Figure 1: Context-aware SV placement case study with `insilicoSV`. a. Samplot illustrations of short-read Illumina and long-read HiFi alignments at homozygous deletion sites placed in a unique/non-repetitive region (top left), exactly overlapping a LINE-1/L1HS repeat (top right), and contained fully within an ALR/Alpha satellite repeat (bottom left). **b.** Precision and recall (reported by Truvari) of short-read and long-read callers stratified by genome context.

Software versions and parameters

The following versions of software tools (and parameters) were used for the case study: Manta 1.6.0 (default parameters), DELLY 0.9.1 (default parameters), Sniffles2 2.2 (default parameters), SVIM 1.4.2 (default parameters), minimap2 2.24 (with `"-ax map-hifi"` preset for mapping HiFi reads and `"-ax map-sr"` preset for Illumina reads; `"-eqx -Y"` for both platforms), DWGSIM 0.1.12-3 (with `"-1 150 -2 150 -y 0 -S 0 -c 0"` and coverage of `"-C 15"` for each haplotype), pbsim3 3.0.1 (with `"-strategy wgs -method sample"`; merged FASTQ file of PacBio HiFi sequencing datasets SRR10382245 and SRR10382249 was provided as the `"-sample"`; `"-depth 15"` was set as the coverage for each haplotype), Truvari 4.2.2 (with `"-pctseq 0 -sizemax=1000000 -passonly -no-ref a"`), samtools 1.6, and samplot 1.3.0.

Data availability

The deletions simulated in each genome context of the case study in section 2.7 are provided as three VCF files at: `https://github.com/PopicLab/insilicoSV/tests/case_study/` and `https://doi.org/10.5281/zenodo.17402009`. These VCFs can be used to recreate the synthetic genomes analyzed in the case study using the `insilicoSV` VCF import feature.
